# Sir Wm. Turner

**Published:** 1916-04

**Authors:** 


**Sir Wm. Turner,** M. D., Principal of the University of Edinburgh, died recently aged 83. He was a physiologist and anatomist, editor of the Journal of Anatomy and Physiology, and a medical writer of note.

